# Spontaneous Renal Hemorrhage Secondary to Acute Pyelonephritis: An Unusual Presentation of Wunderlich Syndrome

**DOI:** 10.7759/cureus.107500

**Published:** 2026-04-21

**Authors:** Angel Portilla, Luis F Ochoa, Vanessa Galvan, Francisco Diaz, Caroline Caraca Lima, Natalia Bezies, Cynthia E Pimentel, Norma A Ortega, Isaac Espinoza, Aurora C Rico, Alejandro Ovando

**Affiliations:** 1 Surgery, Hospital General ISSSTE (Instituto de Seguridad y Servicios Sociales de los Trabajadores del Estado) Presidente General Lázaro Cárdenas, Chihuahua, MEX; 2 Surgery, IMSS (Instituto Mexicano del Seguro Social) Regional General Hospital No. 1, Ciudad Obregon, MEX; 3 School of Medicine, Universidad Autonoma de Baja California, Mexicali, MEX; 4 School of Medicine, Universidad Xochicalco, Mexicali, MEX; 5 Surgery, Hospital de Alta Especialidad de Veracruz, Veracruz, MEX; 6 Surgery, Hospital Regional de Alta Especialidad Zumpango, Zumpango, MEX

**Keywords:** non-obstructive pyelonephritis, renal pathology, spontaneous renal hemorrhage, spontaneus, wunderlich syndrome

## Abstract

Wunderlich syndrome (WS) is a critical urological emergency defined by spontaneous, non-traumatic renal hemorrhage into the subcapsular and perirenal spaces. While typically associated with renal neoplasms like angiomyolipoma or renal cell carcinoma, this report describes an uncommon presentation of WS secondary to acute pyelonephritis. This case highlights the diagnostic challenge of differentiating infectious etiologies from ruptured malignancies in the acute setting. A 50-year-old female patient with a history of long-standing rheumatoid arthritis presented with sudden-onset, severe left lumbar pain, nausea, and vomiting. Physical examination revealed a palpable left flank mass and tenderness. Laboratory studies were significant for neutrophilic leukocytosis (14,010 µL; 89% neutrophils) and abundant bacteriuria. An abdominal CT scan demonstrated a large (10 x 8.5 x 13.5 cm) hyperdense posterolateral collection within the left kidney with loss of normal renal morphology. Due to active hemorrhage and the intraoperative identification of multiple suspicious parenchymal nodules, an emergency total left nephrectomy was performed. Surprisingly, histopathological evaluation revealed no evidence of neoplasia, confirming a diagnosis of acute pyelonephritis with microabscess formation and associated subcapsular hematomas. The patient was stabilized postoperatively with blood transfusion and discharged in stable condition. Although WS is predominantly neoplasic in origin, acute infectious processes can precipitate catastrophic renal hemorrhage. This case underscores that emergency nephrectomy remains a justified, life-saving intervention when hemodynamic stability is compromised, or malignancy cannot be excluded, even in the presence of purely infectious triggers.

## Introduction

Wunderlich syndrome (WS) represents a critical diagnostic frontier in emergency urology, defined by spontaneous, non-traumatic hemorrhage into the subcapsular and perirenal spaces [1]. This clinical entity demands rapid identification to prevent catastrophic hemodynamic collapse, as the retroperitoneal space can sequester significant blood volumes before systemic signs appear [2]. In simple terms, this condition involves bleeding that occurs around the kidney, either beneath its outer capsule or within the surrounding fatty tissue, which can rapidly accumulate and compress adjacent structures before becoming clinically evident. Although the condition was first described in the 18th century, it was Carl Reinhold August Wunderlich who, in 1856, codified the clinical presentation that remains a cornerstone of surgical semiology [3,4]. However, modern clinical audits reveal that the classical Lenk’s triad, comprising acute flank pain, a palpable loin mass, and hypovolemic shock, is strikingly absent in over 80% of contemporary patients, often leading to initial diagnostic obfuscation [5].

The etiological spectrum of WS is predominantly dominated by renal neoplasms, which account for approximately 60% to 65% of documented cases [6]. Among these, angiomyolipoma (AML) stands as the most prevalent benign driver, particularly when lesions exceed the critical diameter of 4 cm, a threshold traditionally associated with increased vascular fragility [7]. Conversely, renal cell carcinoma (RCC) remains the primary malignant precursor, often presenting as an occult rupture [8]. Beyond oncology, the modern clinician must navigate an expanding list of provocateurs, including the rising incidence of complications secondary to systemic anticoagulation and the vascular stressors of end-stage renal disease (ESRD) [9]. In the era of precision medicine, the management of WS has undergone a paradigm shift from mandatory emergency nephrectomy to nephron-sparing strategies facilitated by advances in interventional radiology [10].

## Case presentation

A 50-year-old female patient presented to the ED with sudden-onset severe pain in the left lumbar region radiating toward the ipsilateral abdominal region, which began two hours earlier. The pain was associated with nausea and one episode of vomiting of gastric contents, as well as general malaise. She had a known history of 20 years of long-standing rheumatoid arthritis treated with methotrexate, prednisone, and tocilizumab, prior surgical history of uterine curettage, laparoscopic cholecystectomy, and abdominoplasty, with no other relevant medical history. She was questioned about urinary symptoms, which she denied. 

On physical examination, the patient was conscious, oriented to time, place, and person, cooperative, with adequate coloration and hydration of mucous membranes and skin. She exhibited a pain-related facial expression. The abdomen was distended due to adipose panniculus; bowel sounds were present but decreased. Deep and mid-level palpation elicited tenderness in the hypogastrium, left flank, and left iliac fossa. A non-mobile abdominal mass was palpated in the left hemiabdomen. Rebound tenderness maneuver in the painful areas was positive. Giordano’s maneuver was also performed and was positive on the left side. The remainder of the physical examination showed no abnormalities.

Laboratory test results are given in Table 1. The presence of leukocytosis due to neutrophilia stood out, along with hyperglycemia that could have been associated with the infectious/inflammatory process, as well as a mild elevation of lactate dehydrogenase (LDH) and uric acid; the remaining laboratory values were within normal ranges. Urinalysis was also performed (Table 2). The most notable findings were the presence of leukocytes, abundant bacteria, and microscopic hematuria, which together are consistent with a urinary tract infection, in addition to the leukocytosis observed in the complete blood count.

**Table 1:** Laboratory tests at admission LDH: lactate dehydrogenase

Parameter	Patient Value	Reference Range
Hemoglobin	14.6 g/dL	12-16
Hematocrit	41.2%	36-48
Platelets	148 ×10³/μL	150-400
Leukocytes	14,010/μL	4,50-11,00
Neutrophils	89%	50-70
Glucose	171 mg/dL	70-110
Creatinin	1.1 mg/dL	0.5-1.1
Blood Urea Nitrogen	18.6 mg/dL	7-20
Uric Acid	6.2 mg/dL	2.6-6
LDH	290 U/L	140-280
Amylase	53 U/L	28-100
Albumin	4.2 g/dL	3.5-5

**Table 2:** Urinalysis at admission

Parameter	Patient Result/Value	Reference Range
Aspect	Turbid	Ambar
Proteins	75 mg/dL	None
Ketones	15 mg/dL	None
Hemoglobin	50 mg/dL	None
Leukocytes	4 per high-power field	None
Erythrocytes	2 per high-power field	None
Calcium oxalate crystals	Scant	None
Bacteria	Abundant	None

**Figure 1:**
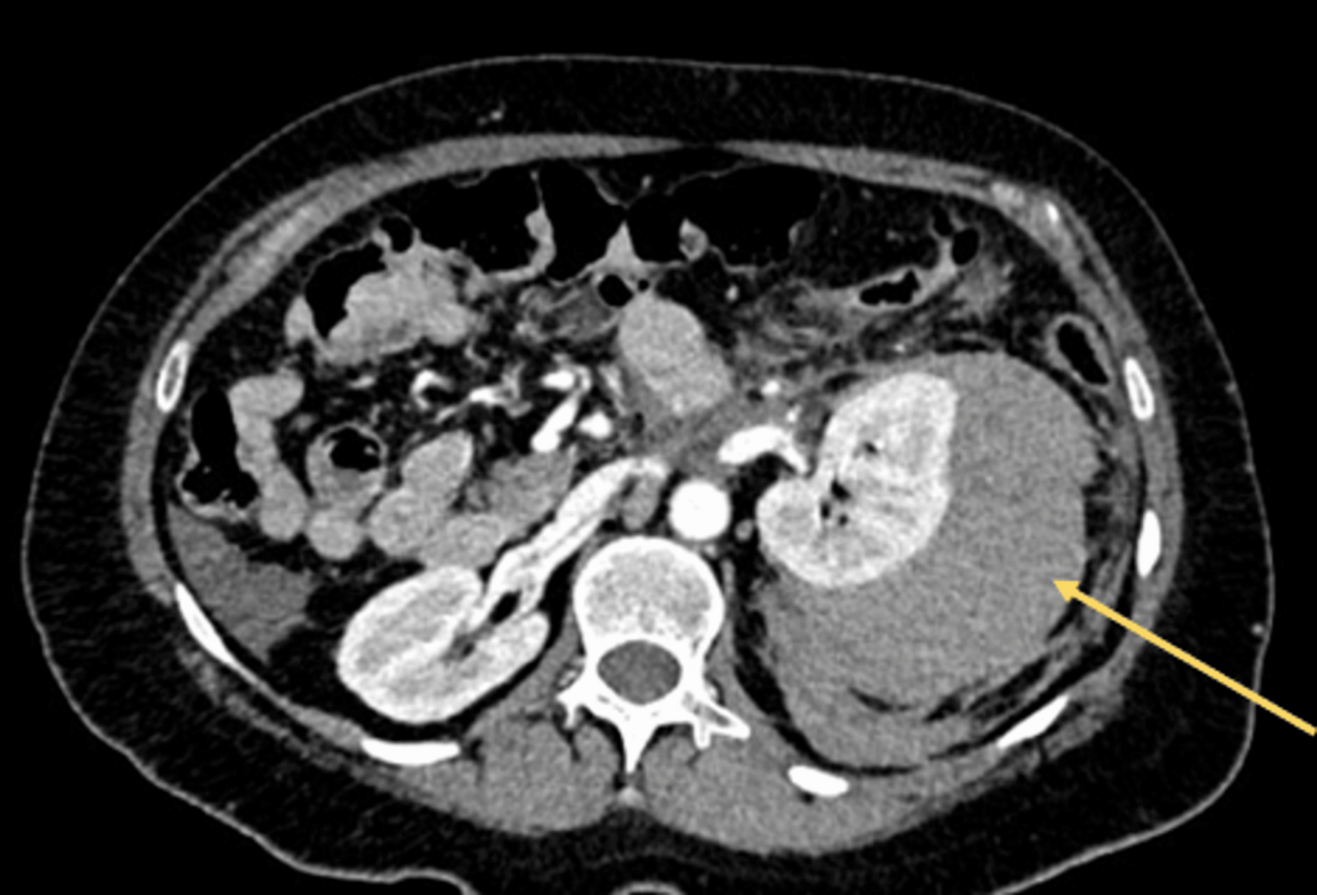
Contrast-enhanced abdominal CT (axial view) Left kidney with a large hyperdense posterolateral collection (yellow arrow). Measurements approximately: 10 × 8.5 × 13.5 cm. Loss of left renal morphology. Obliteration of the perirenal fat stranding. No adjacent mass is observed.

**Figure 2:**
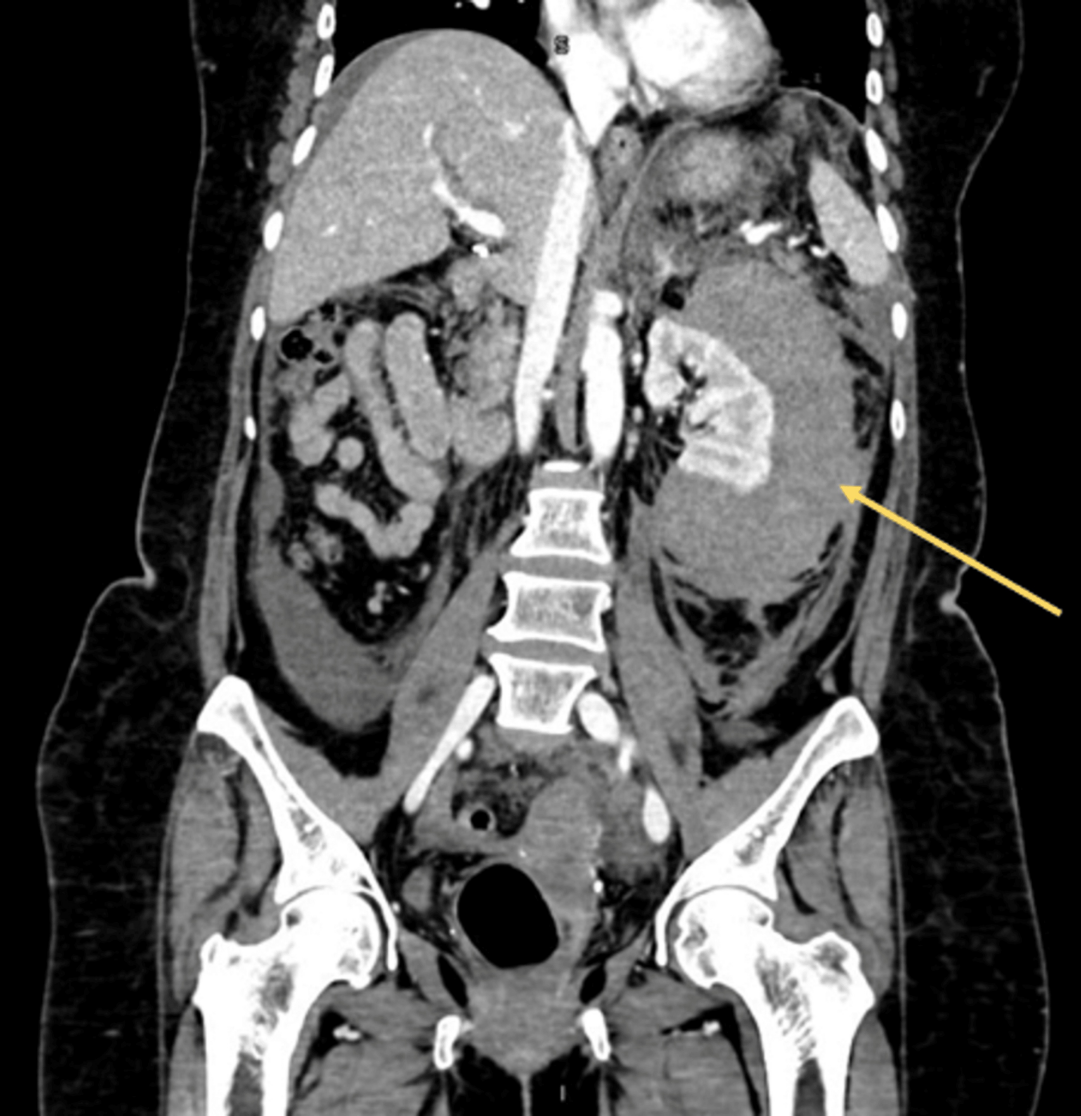
Contrast-enhanced abdominal CT (coronal view) Left kidney with a large hyperdense posterolateral collection (yellow arrow). Measurements approximately: 10 × 8.5 × 13.5 cm. Loss of left renal morphology. Obliteration of the perirenal fat stranding. No adjacent mass is observed.

**Figure 3:**
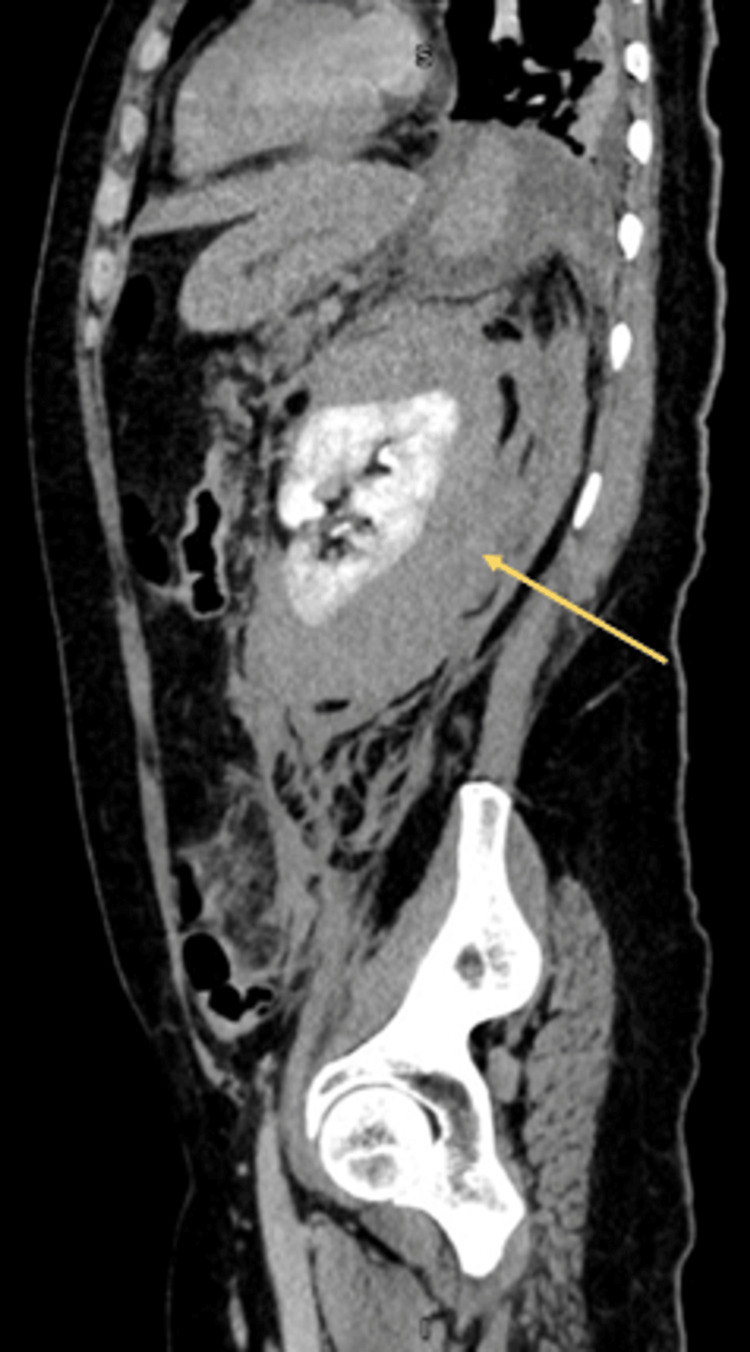
Contrast-enhanced abdominal CT (sagital view) Left kidney with a large hyperdense posterolateral collection (yellow arrow). Measurements approximately: 10 × 8.5 × 13.5 cm. Loss of left renal morphology. Obliteration of the perirenal fat stranding. No adjacent mass is observed.

**Figure 4:**
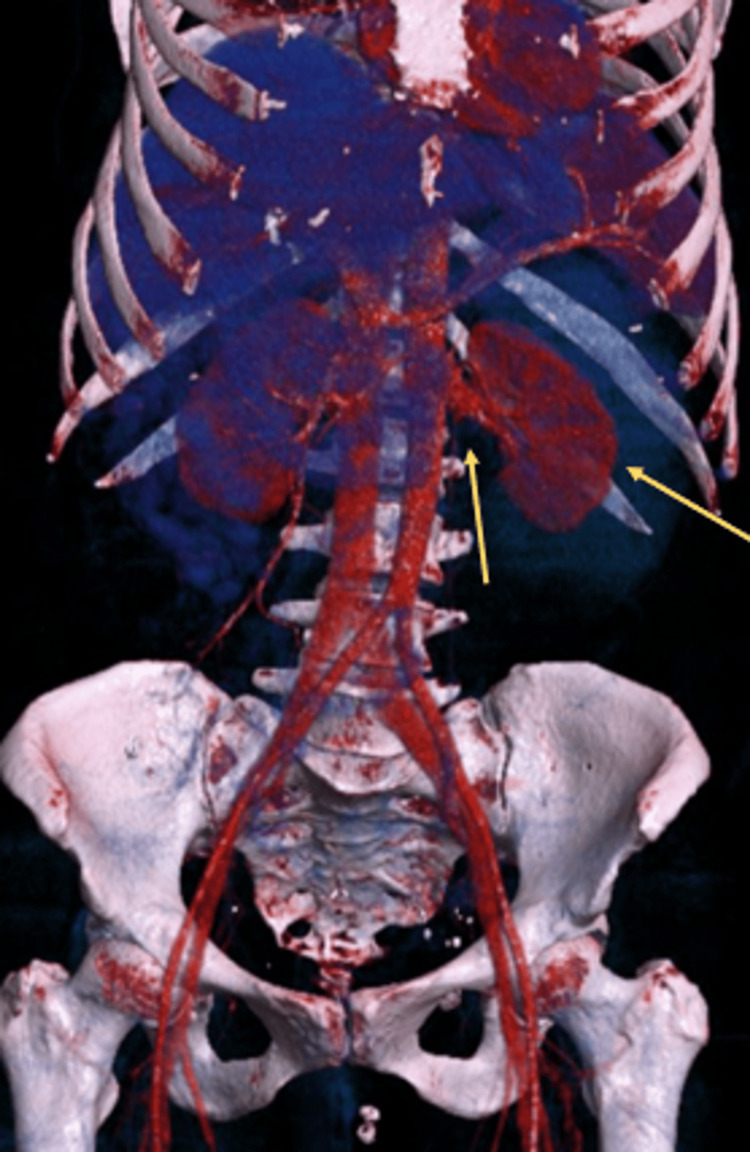
Contrast-enhanced abdominal CT (3D reconstruction) No vascular malformations or specific bleeding source are identified (yellow arrow).

Following the completion of laboratory tests and urinalysis, a contrast-enhanced abdominal CT scan was performed (Figures 1-4), showing the left kidney with a large hyperdense posterolateral collection, approximately 10 × 8.5 × 13.5 cm. There was loss of left renal morphology and obliteration of the perirenal fat stranding. No adjacent mass was observed. Three-dimensional (3D) CT showed no vascular malformations or a specific bleeding source.

Due to the clinical presentation, laboratory findings, and imaging results, the attending physician considered several differential diagnoses: (i) renal abscess, supported by leukocytosis, 89% neutrophilia, abundant bacteriuria, and a large collection, along with the presence of chronic immunosuppression, (ii) spontaneous renal hematoma, favored by hyperdensity in CT scan, abrupt onset of maximal-intensity pain, absence of fever, and no identified trauma*, *and (iii) complicated renal tumor, primary renal neoplasm with spontaneous hemorrhage or abscess formation must be ruled out. 

The patient was taken urgently to the operating room and placed in the right lateral supine position. Asepsis and antisepsis of the thoracolumbar and abdominal regions were performed, and sterile drapes were placed. A left lumbotomy incision was made as the surgical approach. Dissection was carried out by planes, confirming hemostasis with electrocautery. The thoracolumbar fascia was identified, incised, and the retroperitoneum was entered, where Gerota’s fascia containing a renal hematoma was observed. The fascia was dissected until completely isolated. It was then incised, and multiple clots were drained, approximately 500 mL in volume.

**Figure 5:**
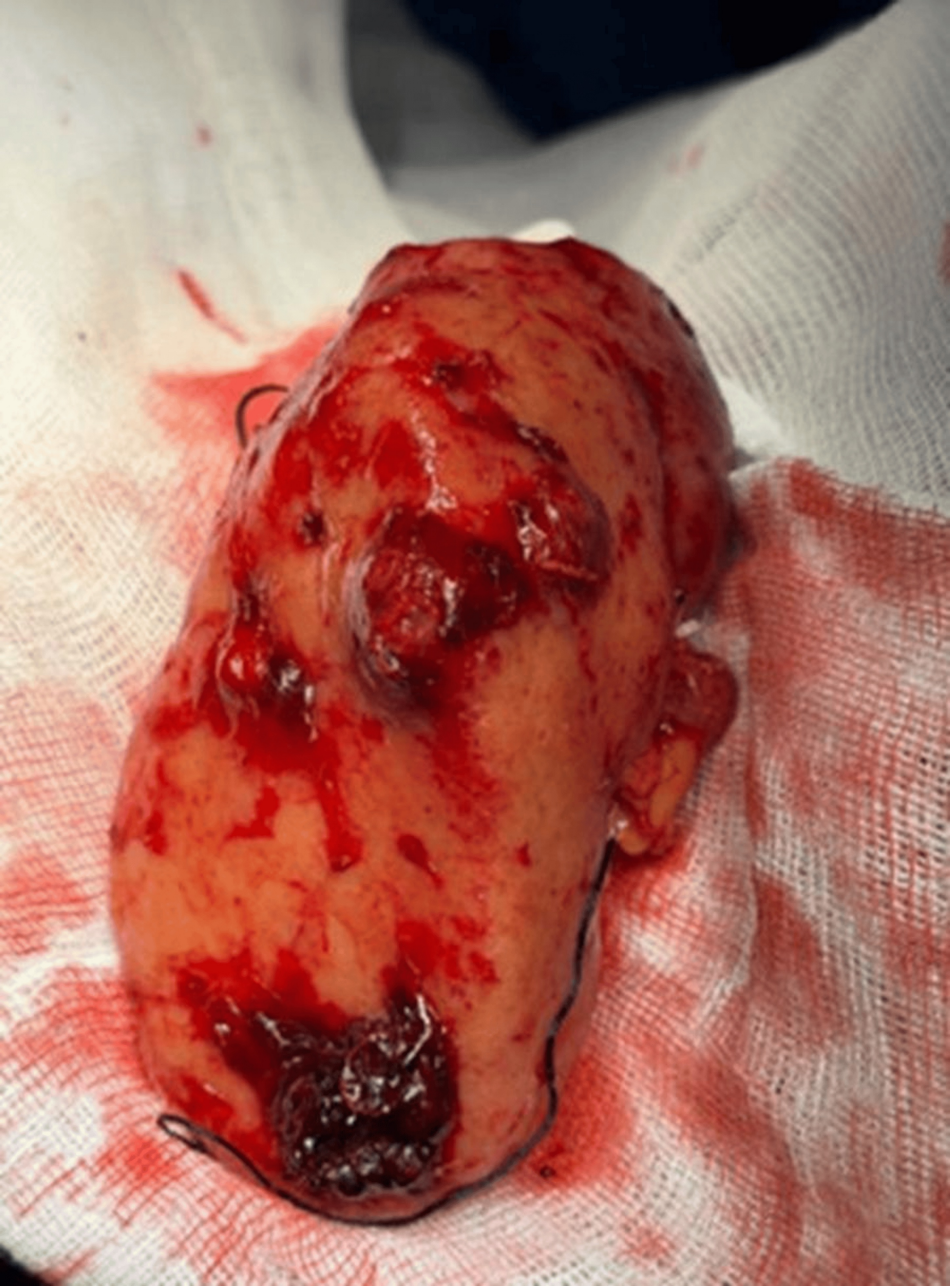
Left kidney after total nephrectomy. Left kidney was observed with multiple tumors and blood clots.

**Figure 6:**
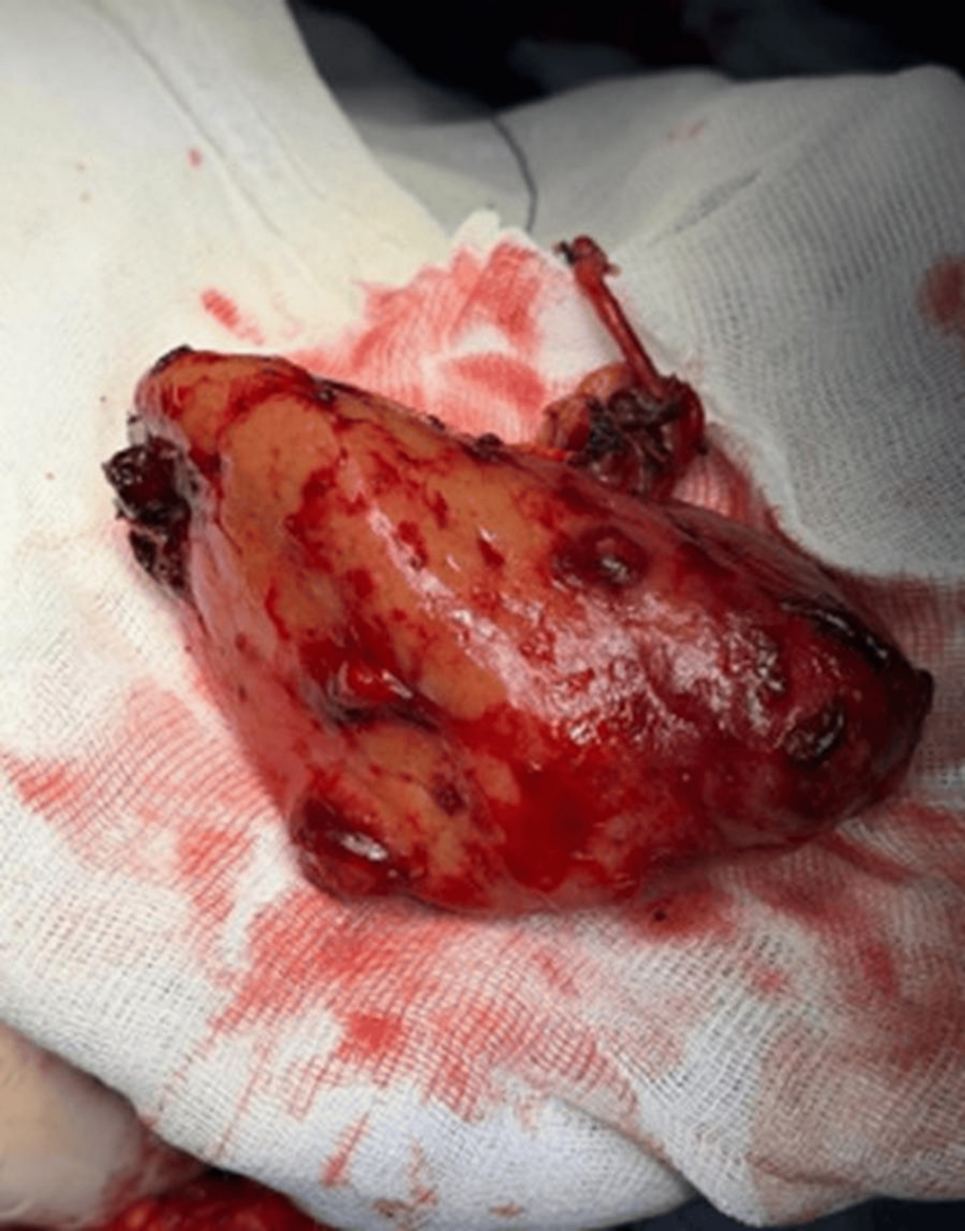
Left kidney after total nephrectomy. Left kidney was observed with multiple tumors and blood clots.

Renal parenchyma was observed with multiple tumors measuring approximately 1.5 cm, the largest being 2 cm in diameter, with active bleeding at both the tumoral and renal parenchymal levels; therefore, a total nephrectomy was decided. Dissection of the renal hilum was continued, identifying two veins and one artery. Two proximal Hem-o-lok clips (Teleflex Incorporated, Wayne, Pennsylvania, United States) were placed on the artery and vein, and one distal clip, after which they were transected. The left kidney was removed (Figures 5, 6). The left ureter was identified and ligated at the middle third with 1-0 silk suture. Hemostasis was verified.

A Penrose drain was placed, exteriorized to the anterior abdominal region, positioned in the renal fossa, and secured to the skin with a simple 2-0 nylon suture. Closure was then performed by layers using 1-0 Vicryl. The subcutaneous cellular tissue was approximated with 2-0 Vicryl, and the skin was closed with interrupted 2-0 nylon sutures.

The surgical procedure was completed with a correct surgical count. Estimated blood loss was 700 mL. During the intraoperative period, transfusion of one unit of packed red blood cells was required. The patient was transferred to recovery in stable condition. The surgical specimen was sent for histopathological study.

**Figure 7:**
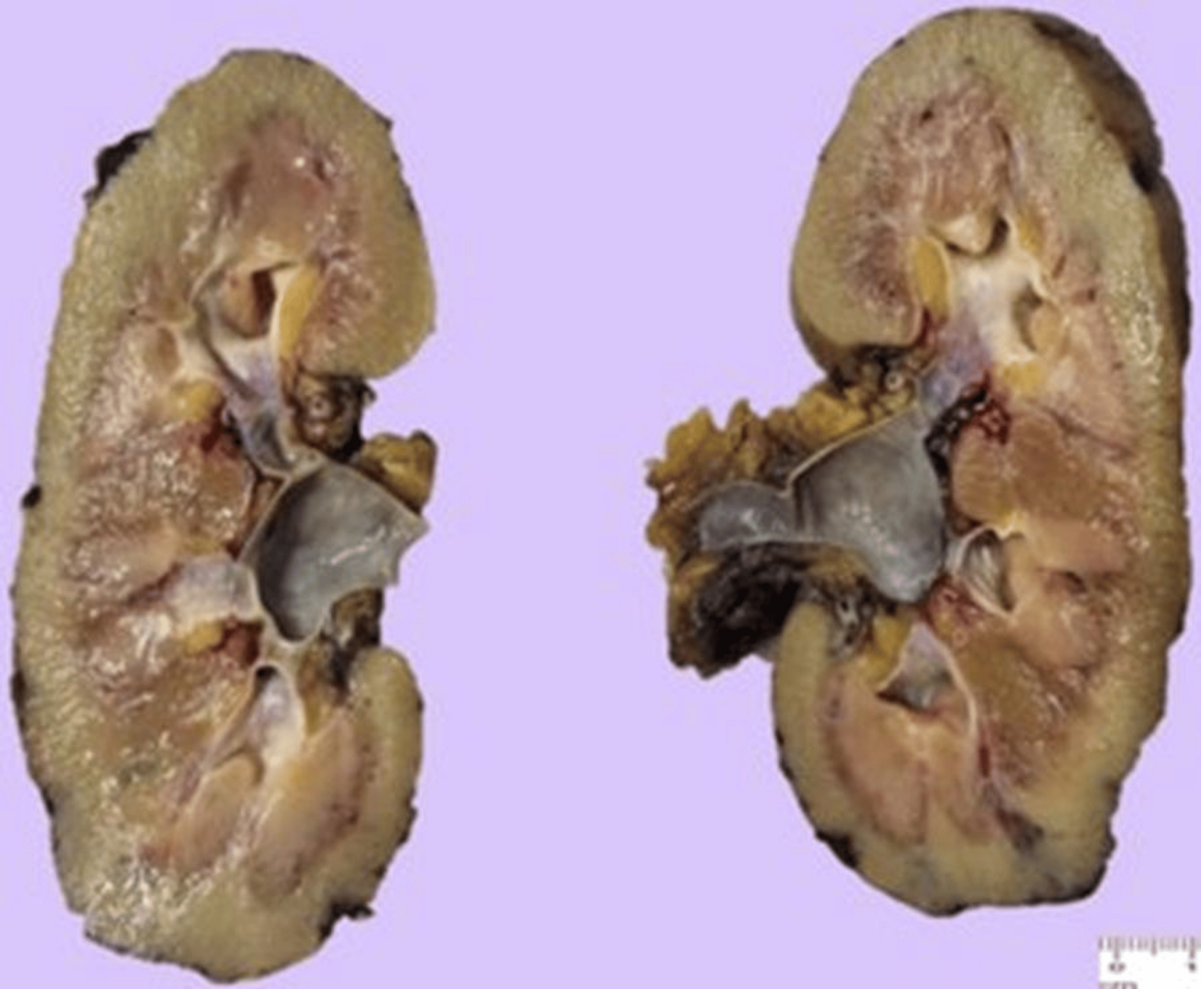
Left kidney sectioned in half.

Histopathology described the left nephrectomy specimen measuring 12.2 × 5.6 × 5 cm. On the capsular surface, hemorrhagic-appearing nodules were identified, measuring up to 0.6 × 1.2 cm. A ureter measuring 5 × 0.6 cm was identified and was patent. On sectioning, the specimen was soft, with regular parenchyma and preserved corticomedullary differentiation (Figure 7). The nodular areas showed a hemorrhagic internal surface, with no additional lesions identified.

**Figure 8:**
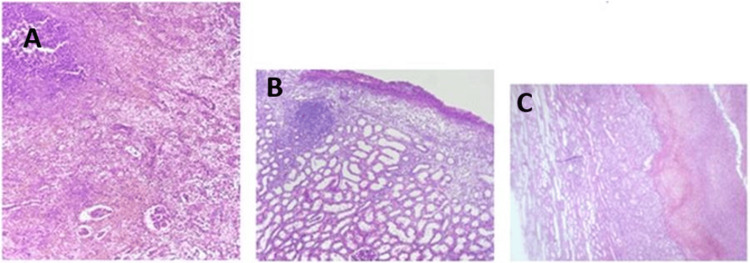
Histological sections (hematoxylin and eosin) of the left kidney A. Superficial nodular areas with subcapsular hematomas and eroded regions. B. Toward the parenchyma, areas/foci of neutrophilic infiltrate and microabscess formation are identified. C. The remaining parenchyma and the ureter show no significant alterations.

Histological sections showed superficial nodular areas with subcapsular hematomas and eroded regions. Toward the parenchyma, areas/foci of neutrophilic infiltrate and microabscess formation were identified. The remaining parenchyma and the ureter showed no significant alterations (Figure 8). According to the histopathological diagnosis, it was acute pyelonephritis with microabscess formation, associated with subcapsular hematomas, without evidence of neoplasia. There was no evidence of renal calculi or malignancy. A viable ureteral margin was observed, without evidence of lesions.

The patient had a favorable clinical course, responding adequately to empirical antibiotic therapy and the prescribed analgesics. She maintained appropriate urine output and showed a decrease in drain output in the subsequent days. She was discharged on postoperative day 3 with an antibiotic regimen, analgesics, wound care instructions, and a follow-up appointment in the urology outpatient clinic.

## Discussion

The clinical presentation of this 50-year-old patient represents a profound diagnostic challenge, as the initial findings pointed toward a ruptured renal neoplasm. While WS is classically associated with the rupture of benign or malignant tumors, our findings revealed a much more insidious etiology: acute pyelonephritis with microabscess formation [2]. The patient's leukocytosis (14,010/μL) and 89% neutrophilia, coupled with abundant bacteriuria, provided the only subtle clues toward an infectious process. However, the presence of a hyperdense collection on CT and the intraoperative visualization of five distinct hemorrhagic nodules created a surgical scenario where malignancy was the most statistically probable and dangerous diagnosis to manage [11].

In the global literature, over 60-65% of WS cases are secondary to renal angiomyolipoma or RCC [6]. Spontaneous hemorrhage secondary to non-specific acute pyelonephritis, as seen in our patient, remains an epidemiological rarity [11]. A 10-year longitudinal analysis confirms that non-neoplastic inflammatory ruptures represent only a fraction of the surgical presentations of WS [12]. Most documented infectious causes of WS involve chronic processes like xanthogranulomatous pyelonephritis; thus, the rapid progression of acute microabscesses to a 500 mL hemoperitoneum is an extraordinary event [10]. Furthermore, the current patient’s history of rheumatoid arthritis may have provided a biological substrate for this severity, as chronic inflammatory states can compromise the structural integrity of the renal microvasculature, predisposing it to rupture under the stress of acute infection; systemic rheumatoid arthritis-related vascular compromise can manifest as increased fragility of small-to-medium vessels, which could have exacerbated the parenchymal erosion and catastrophic hemorrhage triggered by the acute microabscesses [13]. The progression of symptoms despite initial stabilization often necessitates a transition from conservative management to surgical intervention. As demonstrated in recent case series, a significant drop in hemoglobin and the inability to exclude malignancy during the hyperacute phase are primary indications for emergency nephrectomy, even when minimally invasive approaches are considered [14].

A critical point of discussion is the decision to perform an emergency radical nephrectomy instead of conservative management or selective arterial embolization (SAE). While SAE is the current "Gold Standard" for hemodynamically stable patients [15], our patient exhibited active parenchymal bleeding and macroscopically suspicious multifocal lesions. The decision to proceed with an emergency radical nephrectomy is further supported by systematic reviews of contemporary management patterns [16]. As noted by Ahn et al., nephrectomy remains the most common definitive intervention in spontaneous renal hemorrhage, particularly when malignancy (RCC) or large symptomatic AML is suspected [16]. In such scenarios, surgical excision provides the necessary balance between acute hemodynamic stabilization and the prevention of oncological progression [2,11].

The clinical resolution of this case offers a profound pedagogical contribution to the management of acute urological emergencies, primarily by illustrating the treacherous mimicry of renal infectious processes. It serves as a stark reminder that acute pyelonephritis, when presenting with microabscess formation, can achieve a radiological and macroscopic profile that is virtually indistinguishable from a ruptured multifocal neoplasm. Consequently, clinicians must maintain an elevated index of suspicion for infectious etiologies in patients presenting with systemic inflammatory markers and comorbid autoimmune conditions, even when cross-sectional imaging strongly suggests a neoplastic origin [17,2]. Table 3 shows the indications for each type of treatment.

**Table 3:** Comparison between open surgery and endovascular treatment References: [1,14,16]

Characteristics	Radical nephrectomy	Conservative management (± embolization)
Main indications	Persistent hemodynamic instability; suspicion of malignancy; uncontrolled hemorrhage; extensive renal rupture	Hemodynamically stable patient; likely benign etiology; contained bleeding
Objective	Definitive control of bleeding and lesion removal	Control of bleeding while preserving the kidney
Hemorrhage control	Immediate and definitive	Generally effective, but may require embolization or reintervention
Etiology management	Definitive (especially in malignant tumors)	May be deferred; requires follow-up and additional interventions
Renal function preservation	No (complete loss of the affected kidney)	Yes (nephron-sparing approach)
Morbidity and mortality	Higher, especially in emergency settings	Lower in selected patients
Risk of rebleeding	Low	Moderate (depending on the cause)
Resource requirements	Operating room, surgical team	Monitoring, blood bank and interventional radiology
Recovery time	Longer	Generally shorter
Current role	Reserved for severe cases or oncologic suspicion	First-line in stable patients

There are inherent diagnostic limitations of CT in the hyperacute phase of WS, where the attenuation values of clotted blood and inflammatory exudate often overlap, obscuring underlying parenchymal details [18,11]. Ultimately, the disparity between the intraoperative suspicion of malignancy and the final histopathological confirmation of a benign, albeit aggressive, infection underscores the fact that surgery in the setting of spontaneous hemorrhage is often a battle for hemodynamic control rather than a quest for pathological certainty [18]. Surgeons should thus interpret a benign postoperative report not as a failure of diagnostic accuracy, but as a validation of the extreme biological variability and destructive potential of renal inflammatory disease [12].

## Conclusions

WS remains a rare but life-threatening urological emergency requiring a high index of clinical suspicion, particularly because its presentation is frequently nonspecific and the classical Lenk’s triad may remain absent. While renal neoplasms remain the primary etiology, this case illustrates that acute inflammatory processes, specifically, pyelonephritis with microabscess formation, can precipitate spontaneous renal hemorrhage, mimicking the clinico-radiological profile of a ruptured malignancy. 

In the acute setting, the limitations of non-contrast CT may preclude definitive etiological differentiation, thereby complicating the surgical decision-making process. Consequently, management strategies must prioritize hemodynamic stabilization over diagnostic certainty. In this context, radical nephrectomy remains a justified, life-saving procedure when faced with active hemorrhage or high suspicion of occult malignancy. Ultimately, this case highlights the necessity of a broad differential diagnosis that incorporates infectious etiologies. In emergency urology, the primary objective of surgical intervention remains the achievement of rapid hemodynamic control, regardless of a definitive preoperative diagnosis.
